# SpreadRank: A Novel Approach for Identifying Influential Spreaders in Complex Networks

**DOI:** 10.3390/e25040637

**Published:** 2023-04-10

**Authors:** Xuejin Zhu, Jie Huang

**Affiliations:** 1 School of Cyber Science and Engineering, Southeast University, Nanjing 211189, China; 2 Purple Mountain Laboratories, Nanjing 211111, China

**Keywords:** information diffusion, node centrality, influential spreaders, complex networks, SIR model

## Abstract

Identifying influential spreaders in complex networks is critical for information spread and malware diffusion suppression. In this paper, we propose a novel influential spreader identification method, called SpreadRank, which considers the path reachability in information spreading and uses its quantitative index as a measure of node spread centrality to obtain the spread influence of a single node. To avoid the overlapping of the influence range of the node spread, this method establishes a dynamic influential node set selection mechanism based on the spread centrality value and the principle of minimizing the maximum connected branch after network segmentation, and it selects a group of nodes with the greatest overall spread influence. Experiments based on the SIR model demonstrate that, compared to other existing methods, the selected influential spreaders of SpreadRank can quickly diffuse or suppress information more effectively.

## 1. Introduction

In real-world scenarios, many interactions between individuals can be modeled as complex networks. For instance, the spread of rumors in social networks [1,2,3], communication networks between users in email systems [4], and the spread of computer viruses [5]. Furthermore, information spread in these networks exhibits certain diffusion patterns, and certain nodes have a greater influence on the spread process. Accurately identifying these influential spreaders and isolating or immunizing a small number of nodes at a limited cost can effectively inhibit the rapid spread of malicious information such as rumors or viruses. Conversely, in scenarios where the goal is to quickly disseminate information throughout a network, such as the promotion of commodity advertisements to an entire social network [6,7], influential spreaders can be used as the initial communicators, maximizing the efficiency of the information spread.

The majority of research on identifying influential spreaders in complex networks employ node centrality measurement methods to score and rank the influence of all nodes in the network. Common classical node centrality measurement methods include degree centrality [8], betweenness centrality [9], k-shell [10], and others. The simplest method is degree centrality, which only relies on the degree of the node and does not consider other factors. Betweenness centrality calculates the frequency with which the shortest distance between all pairs of nodes passes through a node. The higher the frequency, the more important the node is. The k-shell method recursively removes nodes whose degree is less than or equal to *k* in the network in each round of operations and assigns a k-score to these removed nodes as *k*. This process is repeated until the k-score values of all nodes are obtained. Higher values indicate that the node is more important. In recent years, many centrality methods have also been developed that combine multiple factors. These methods also consider network topology information, such as node degree, clustering coefficient, and node neighbors [11,12,13,14,15,16,17,18,19]. Keyou et al. proposed deterministic algorithms, which converge in finite time, for the distributed computation of the degree, closeness, and betweenness centrality measures in directed graphs [20]. Gong et al. proposed a memetic algorithm for community-based influence maximization in social networks [21]. Additionally, some researchers have studied node centrality for specific scenarios. Google proposed a method called PageRank for determining the importance of internet pages, which considers the number of connections between different pages and the weights of neighbor nodes [22].

The method of identifying influential spreaders based on node centrality is limited to evaluating the spread ability of individual nodes and cannot accommodate scenarios that require the immunization of a group of nodes. For instance, in the context of computer virus propagation, it is often necessary to select a group of critical nodes for immune operations to suppress the spread. Merely selecting the top-k nodes based on the ranking results for immunization may result in overlapping influence scopes between nodes, leading to poor overall immunization effectiveness. Consequently, the academic community has developed various node set influence identification methods, which aim to assess the influence range between different nodes. If the scopes of influence of two nodes significantly overlap, the selection probability of one of the nodes is reduced to determine the node set that can achieve the overall immune effect. Zhang et al. [23] proposed a simple and effective iterative method named VoteRank to identify a set of decentralized communicators with good spreading ability. In this approach, nodes vote on their neighbors in each round, and the voting power of the neighbors of the elected seed node will decrease in subsequent rounds. Inspired by k-shell, Jiang et al. [24] transformed the node selection problem into the problem of finding densely connected groups in the network, and then proposed a heuristic group discovery (HGD) algorithm, which can perform well in large social networks. Additionally, Kimura et al. proposed the SPM and SP1M models, based on the independent cascade model, for natural social network information spread. This model provides a more precise estimation of the influence of the selected node set [25].

The method of node centrality used in the aforementioned approach does not account for specific factors and conditions in information spread scenarios, such as the diversity of spread paths and spread distance. These factors determine the difficulty of nodes spreading information to other nodes in the network. Additionally, in the process of selecting the node set, we need to consider the maximum connected branch of the remaining node network in order to minimize the maximum diffusion scale of the remaining nodes and create the network isolation to block the spread. This consideration has been discussed in previous studies [26,27,28].

To address these issues, this paper proposes a new method, called SpreadRank, for identifying influential spreaders in complex networks. The method is designed specifically for information spread scenarios and takes into account the aforementioned factors. The main contributions of this paper are as follows:

(1) This paper introduces a novel approach for measuring node centrality for information spread. Our method computes the spread centrality value of each node by assessing its path reachability to local vital nodes. Nodes that are more easily reached from other local vital nodes are assigned a higher spread centrality value, indicating greater spread potential. The path reachability is determined by factors such as path distance, path diversity, and number of paths.
(2) Furthermore, we propose a method for selecting influential spread node sets based on dynamic selection. In this iterative process, the spread centrality value of nearby nodes is dynamically updated, and nodes are added to the influential node set based on their size until the maximum connected component meets the threshold setting percentage. Less influential nodes are subsequently removed, resulting in the minimum number of influential spread nodes that meet the threshold ratio.
(3) Through experiments using the SIR spread model on real network datasets, we demonstrate that our proposed SpreadRank method has greater spread influence compared to other existing common identification methods.

The rest of this article is organized as follows. The Section 2 introduces the node influence evaluation method, SpreadRank, proposed in this paper in detail, including the calculation of node spread centrality and the selection of influential spread node sets. Section 3 verifies the effectiveness of the method through experiments. Section 4 presents the conclusions and future research directions.

## 2. Materials and Methods

This section will introduce the detailed process of the proposed SpreadRank method.

### 2.1. Node Spread Centrality Based on Path Reachability

Given a network G=(V,E), *V* represents the set of nodes in the network, and *E* represents the set of edges. Let N=|V| be the total number of network nodes. $e_{ij}$ represents the edge connecting node *i* and node *j*; $n_{i}$ represents the neighbor node set of node *i*; and $d_{i}$ represents the number of links of node *i*, that is, the degree of node *i*.

**Figure 1:**
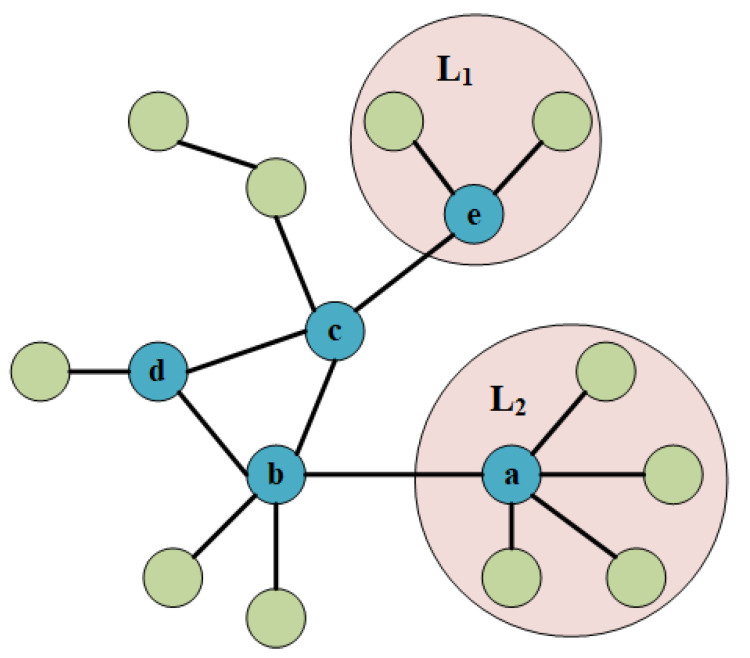
Cross-local spread of information. The path reachability between local vital nodes *a* and *e* determines the information spread between area $L_{1}$ and area $L_{2}$.

In the information spread process in complex networks, nodes with higher degrees have a strong spreading ability in the local range near them, so we call them local vital nodes. Real networks often have scale-free characteristics, that is, the degrees of most nodes are lower than the average degree; therefore, the local vital nodes selected in this paper are nodes with degrees greater than the average degree of the network. In addition, whether a message can be transmitted from one local region to another distant local region in the network depends on the reachability of the spread path between the local vital nodes corresponding to these two regions. We call this spread path reachability. As shown in Figure 1, there are 5 local vital nodes in the network. Whether the information from the local region $L_{2}$ can be transmitted to $L_{1}$ depends on the path reachability between the local vital nodes *a* and *e*. A higher reachability implies an easier spread of information across the network. Therefore, this paper proposes the path reachability index as a measure of node spread ability. If the path reachability index from a node to all other nodes is higher, the node’s spread ability is stronger. The spread centrality value of each node is computed by calculating the overall path reachability index from that node to all other local vital nodes, and then ranking all local vital nodes based on their spread ability.

The path reachability index proposed in this paper comprises three factors: path length (PL), path number (PN), and path diversity (PD), as summarized in Table 1. Shorter path lengths between two nodes facilitate the spread of information to the target node. Moreover, increasing the number of paths between two nodes can help the original message spread to the target node through different paths. Path diversity is defined as the degree of noncoincidence between all spread paths between two nodes, as per Definition 1. High coincidence between spread paths, i.e., passing through multiple identical nodes, can lead to disconnection of all spread paths at the immune node. In contrast, spread paths with low similarity, i.e., high diversity, can enable information to spread to the target node in different directions.

It is important to note that in networks with a high average degree of nodes, finding all paths between two nodes can be computationally expensive. However, in line with the small-world characteristics of real networks, it is generally accepted that there is a six-degree theory between any two nodes [29,30]. This means that two nodes can typically reach each other within six hops. As a result, the source node does not typically require a long path to spread information to the destination node. Therefore, this paper considers the path with PL⩽6 between local vital nodes as an effective path, while ignoring overlong and invalid paths.

**Definition** **1.** *Suppose there are k valid paths between nodes i and j in the network G, then the path diversity $PD_{ij}$ between the two nodes is defined as the mean value of the noncoincidence of the nodes passed between any two valid paths, and its calculation formula is as follows:*

(1) $$PD_{ij}=\frac{1}{C_{2}^{k}}\sum_{m=1}^{C_{2}^{k}}\left(1-\frac{2w_{m}}{d_{m1}+d_{m2}}\right),$$

*where $C_{2}^{k}$ represents the total number of ways to take two paths from *k* paths; $d_{m1}$ and $d_{m2}$ represent the length of two paths of taking the *m*-th way, respectively; and $w_{m}$ indicates the number of coincident nodes of the two paths of the *m*-th way. It should be noted that the approach used to calculate path diversity in this paper bears similarities to the well-known Jaccard distance, which is computed as the ratio of the intersection of two sets to their union.*

If there are *l* local vital nodes in the network, according to formula (1), we can further calculate the path diversity $PD_{i}$ of the local vital node *i* in the network, which indicates the path diversity of the information sent by the node to all other local vital nodes. The calculation formula is as follows:

(2) $$PD_{i}=\frac{1}{l}\sum_{j=1}^{l}PD_{ij}.$$

Similarly, the calculation formulas of the spread path length $PL_{i}$ and the number of spread paths $PN_{i}$ from node *i* to other local vital nodes are:

(3) $$PL_{i}=\frac{1}{kl}\sum_{j=1}^{l}\sum_{m=1}^{k}PL_{ij}^{m},$$

(4) $$PN_{i}=\frac{1}{l}\sum_{j=1}^{l}PN_{ij},$$

where $PL_{ij}^{m}$ represents the length of the *m*-th path between nodes *i* and *j*, and $PN_{ij}$ represents the number of paths between nodes *i* and *j*.

Finally, we integrated the three factors of path length, path number, and path diversity and obtained the path reachability index of each local vital node as its spread centrality value SC. The calculation formula is as follows:

(5) $$SC_{i}=\left[norm\left(PD_{i}\right)+norm\left(PN_{i}\right)+norm\left(\frac{1}{PL_{i}}\right)\right]/3,$$

where $norm\left(\cdot \right)$ is a standard normalization function, which normalizes the result to [0,1], and is determined by the following equation:

(6) $$norm\left(x\right)=\frac{x-\min}{\max-\min},$$

where min and max refer to the minimum and maximum values in that group of data, respectively.

All local vital nodes are sorted according to the spread centrality value SC, and the spread centrality measure of a single node in the network is obtained.

### 2.2. Influential Node Set Based on Dynamic Selection

In the previously mentioned measurement results of node spread centrality, only individual nodes were ranked according to their spread capabilities. However, when these nodes with strong spread ability are combined, the overall spread effect may be unsatisfactory due to significant overlap in the scope of the spread influence between the nodes with high spread centrality. Thus, this paper proposes an influential node set selection method based on the node spread centrality method to achieve the best overall network spread or immune effect. In the following section, we introduce the dynamic influential node set selection method proposed in this paper.

It is evident that nodes with high spread centrality values tend to have a higher overlap in their spread influence as they are closer in distance. Therefore, even if a node has a high spread centrality value, the probability of its selection should be reduced if it is close to other nodes with high centrality values. To address this issue, this paper proposes a spread centrality attenuation method for neighbor nodes. This method considers both the neighbor nodes of the selected node and the second-order neighbors (i.e., the neighbor nodes of neighbors). Specifically, when a node is initially selected as an influential node, the spread centrality values of its surrounding nodes are updated, and the attenuation amplitudes of all its neighbor nodes and second-order neighbor nodes are, respectively, $\frac{1}{2}$ and $\frac{1}{4}$ of the original centrality value.

**Figure 2:**
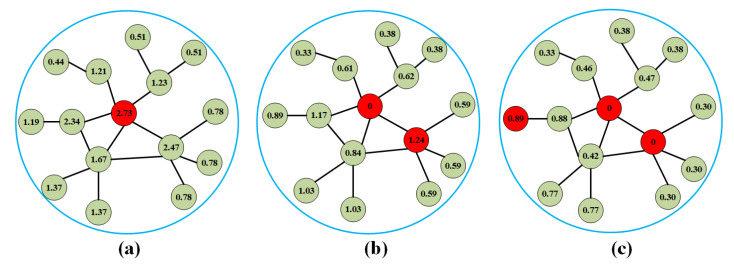
Node spread centrality attenuation method. (**a**) The initial state of network. (**b**) The second round of spread centrality value updates. (**c**) The third round of spread centrality value updates.

Next, we will illustrate the neighbor spread centrality attenuation method. As shown in the initial state of Figure 2a, the maximum node spread centrality value is 2.73, so this node is marked as a preselected influential node. In the second round of spread centrality value updates, as shown in Figure 2b, the value of the preselected influential node in the previous round is reset to 0, the spread centrality of its neighbor nodes is attenuated to half of the original value, and the second-order neighbor nodes are attenuated to the original value of $\frac{3}{4}$. Then, the node with the largest spread centrality value in the network is preselected as the second influential node. By following this process, non-neighbor nodes will be preselected as influential nodes after the third round of node spread centrality value updates, which effectively reduces the coincidence of spread influence of influential nodes, as shown in Figure 2c.

On the other hand, when the most influential set of high-influence nodes is removed from the network, the remaining network will be fragmented into multiple connected branches. The size of the largest connected branch can reflect the effect of network segmentation. The smaller the size of the largest connected branch, the more significant the role of the node set becomes [26]. In the context of immune operations, if the size of the largest connected branch is small enough, malicious information can only be propagated within the smaller connected branch, which can effectively isolate the spread. In this paper, we aim to identify a set of the most influential nodes in the network that is as small as possible. Removing this set of nodes from the network can cause the network to fragment into a large number of sufficiently small branch fragments. Its specific mathematical description is as follows: for a given network *G* with a scale of *N*, assuming that its core node set is *S*, the size of the maximum connected branch of the remaining network $G_{V-S}$ after deleting the set *S* is recorded as $\left|G_{V-S}\right|$, then:

(7) $$q=\frac{\left|G_{V-S}\right|}{N}<\mu ,$$

where *q* represents the relative size of the maximum connected branch, and μ is the threshold for judging whether the network is destroyed as much as possible. In the related literature of influential node discovery algorithms [27,28], it is generally taken that μ=0.01. This paper also adopts the same threshold.

The process of developing an influential node set based on dynamic selection can be divided into two stages: node addition and node deletion, as shown in Algorithm 1.
**Algorithm** **1:** Dynamic selection of influential node set
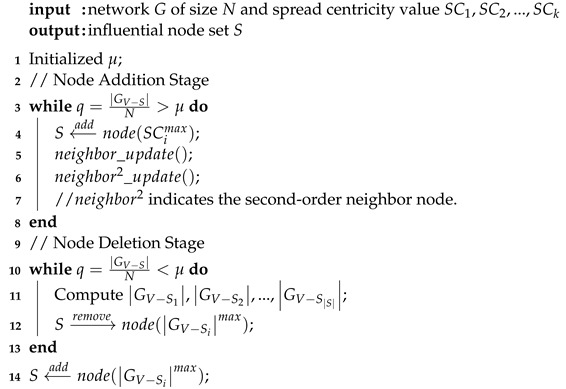

- **Node Addition Stage**: The proposed influential node set selection method in this paper starts with selecting the local vital node with the highest spread centrality value in the network and updating the spread centrality values of its nearby nodes using the neighbor node spread centrality attenuation method. The preselected influential node set is then removed from the network, and the size of the maximum connected branch of the remaining network is checked. If the size of the maximum connected branch is larger than the threshold μ, the process continues with selecting the node with the next highest spread centrality value and adding it to the preselected influential node set. This process repeats until the maximum connected branch size is smaller than the threshold μ.
- **Node Deletion Stage**: Selecting from the preselected influential node set and deleting the node that has the least impact on the size of the maximum connected branch after being put back into the network. Specifically, we record the influential node set of deleted node *i* as $S_{i}$, which is calculated as the maximum value of $\left|G_{V-S_{i}}\right|$. We delete the corresponding node from the preselected influential node set and calculate whether the maximum connected branch size is less than the threshold μ. If it is satisfied, we continue to delete the next node from the preselected influential node set, and cycle in turn until the maximum connected branch size is greater than the threshold μ. The preselected influential node set from the previous round is taken as the final influential node set *S*.

### 2.3. Time Complexity Analysis

In this section, we will analyze the time complexity of the proposed SpreadRank method. In the calculation of spreading centrality, we need to search for effective spreading paths between local vital nodes in the network, which correspond to all paths with a length of less than six, resulting in a time complexity of $O(N^{2})$. Moreover, the calculation of path diversity and path length for all local vital nodes is O(N), so the time complexity of the spreading centrality calculation is $O(N^{2})$. During the dynamic selection of influential node sets, both the node addition and deletion phases require traversing all candidate node sets, with a time complexity of O(N) each. Therefore, the overall time complexity of the SpreadRank method is $O(N^{2})$.

## 3. Results

In order to verify the effectiveness of SpreadRank, the method proposed in this paper, this section presents the results of various experiments conducted on public real network datasets. Well-known methods, such as degree, betweenness, k-shell, VoteRank, and Eigenvector, are selected as comparison benchmarks.

### 3.1. Spread Model

Like other related literature, the SIR epidemic model is used in our experiments to measure the spread efficiency of the nodes. The SIR model is a simple but effective epidemiological model used to describe the spread of infectious diseases in a population, which later has been extended to study information diffusion. A node in the SIR model can be in three states:

- Susceptible (S): the initial state of the node—in a healthy state, but easily infected by viruses;
- Infected (I): infected by a virus, and there is a certain probability of converting to a recovery state;
- Recovery or removal (R): after the infected node is repaired, it becomes immune and cannot be infected again, which is equivalent to being removed from the system.

The SIR model assumes that contact between individuals in the population is random, and that the probability of infection is the same for all individuals. Additionally, infected individuals become immune to the disease and can only transmit the virus to susceptible individuals. Recovered individuals cannot infect others and are also immune to the disease. Based on these assumptions, the SIR model can describe the spread of infectious diseases in a population and predict the trends of virus transmission.

In a complex network, a certain number of initial infected nodes are designated, and the nodes connected to these infected nodes have a probability β of becoming infected in each time step. Simultaneously, the infected node has a probability γ of recovering within the time step. As the spread continues, the infected nodes in the network will eventually disappear, leaving behind only susceptible and recovered nodes. The proportion of recovered nodes indicates the scale of the network that was infected throughout the spread cycle, denoted as $P_{r}$ in this paper. Therefore, we can select a set of influential nodes *S* as the initial infected nodes, and the greater the proportion of recovered nodes in the final system $P_{r}$, the more effective the spread of this group of influential nodes.

The SIR epidemiological model comprises two crucial variables: the infection rate β and the recovery rate γ. These variables have opposite effects on the rate of the information spread. Specifically, the larger the infection rate β, the faster the information spreads, and the larger the recovery rate γ, the more suppressed the information spread becomes. To simulate the SIR model, this paper sets the recovery rate γ to a fixed value of 0.01 and analyzes the experimental results by varying the infection rate β. Moreover, to ensure the validity of the experiment, all the results are based on 100 independent experiments and averaged.

### 3.2. Experimental Setup and Datasets

In this paper, we used the Matlab simulation platform to construct complex network topologies in real-world datasets and selected a set of influential nodes. The experiments were conducted on a computer running the Windows 11 operating system, with a Core i5-9300H CPU and 16 GB memory. To fully verify the effectiveness of the proposed method, it is applied to four real networks with different sizes [31].

- Email: It is an email network of the University at Rovirai Virgili. Each node represents a user, and an edge indicates that two users have an email exchange.
- PGP: The topology of the Gnutella peer-to-peer network. Nodes represent hosts in the Gnutella network, and edges represent connections between the Gnutella hosts.
- Ia-reality: Reality-mining network data consisting of human mobile phone call events between a small set of core users at the Massachusetts Institute of Technology (MIT). Each node represents a user, and an edge indicates a phone call or voicemail between two users.
- DBLP: This is the citation network of DBLP, a database of scientific publications such as papers and books. Each node in the network is a publication, and each edge represents a citation of a publication by another publication.

The basic statistical information of the four real datasets used in this study is presented in Table 2. It can be observed that the real networks differ significantly in terms of node size, average degree, clustering coefficient, and average distance of the shortest path, and these parameters have a certain impact on the information spread [12,13,16,17,18]. The selection of networks with different parameters ensures the generalizability of the proposed SpreadRank method. The email network is the smallest in size, but it has a high clustering coefficient and average degree, indicating that the nodes are closely interconnected. In contrast, the DBLP network is the largest in size, while the PGP network is a server–host network with a relatively sparse node distribution, resulting in a lower clustering coefficient. Furthermore, the Ia-reality network is a sparsely connected network with a low average degree, which is in contrast to the email network.

### 3.3. The Proportion of Spread under Different Infection Rates

**Figure 3:**
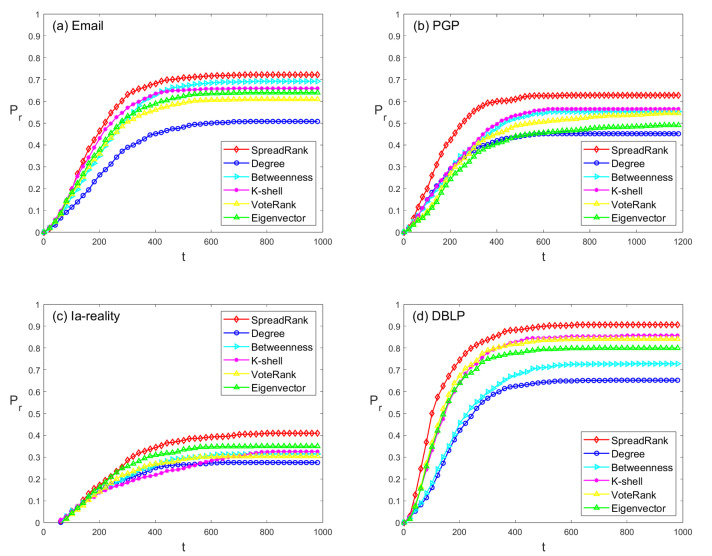
Recovery proportion $P_{r}$ with respect to the time *t* on four datasets in the SIR model. β=0.05; γ=0.01.

We compared the effectiveness of degree, betweenness, k-shell, VoteRank, Eigenvector, and the proposed SpreadRank methods in the above real networks in the SIR model. Figure 3 shows the proportion of recovered nodes for each real network as a function of time steps under a fixed infection rate (β=0.05) and recovery rate (γ=0.01). It can be seen that the SpreadRank method proposed in this paper can spread faster at the initial stage of infection than other methods, and the final infection scale is also larger.

**Figure 4:**
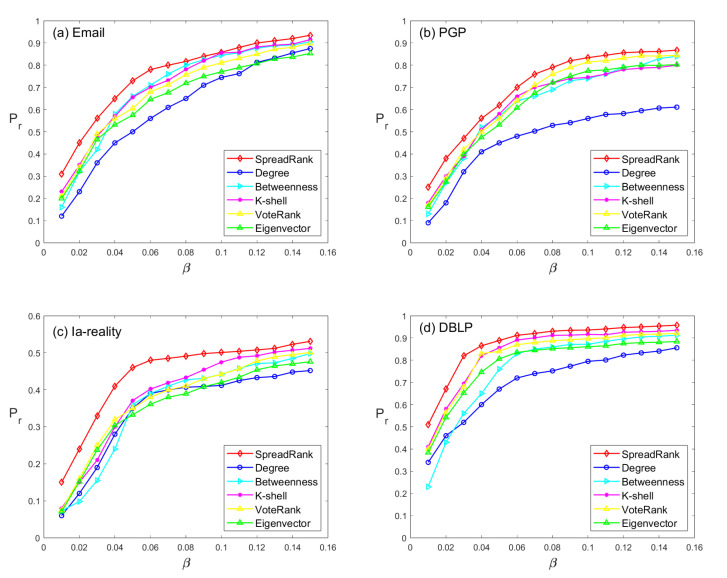
Comparison of the spreading influence of our method with other methods on four datasets in the SIR model with γ=0.01. The influential spreaders were set as the initial infected node.

Figure 4 depicts the change in the final recovery proportion $P_{r}$ with respect to the infection rate β, where β ranges from 0.01 to 0.15, which covers the majority of network information propagation scenarios in practical settings [10,32,33]. The results show that SpreadRank outperforms other methods as it achieves a higher proportion of recovered nodes in the network, which indicates that the selected node set has greater spread influence. Specifically, SpreadRank yields a significantly higher $P_{r}$ than other methods for low infection rates (β<0.04). Moreover, the curve slope of SpreadRank when β is low indicates that it can quickly reach a higher level of information dissemination with an increase in β. Notably, since the average degree of the Ia-reality dataset is low, it can be viewed as a sparsely connected network. As shown in Figure 4c, our method outperforms other methods, implying that SpreadRank has more advantages in sparse networks.

**Figure 5:**
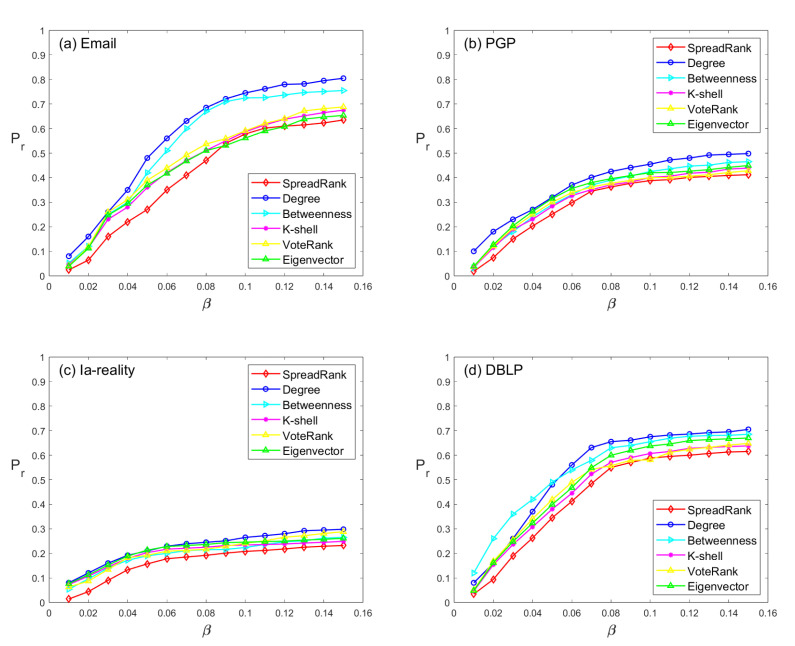
Comparison of the spread suppression capability of our method with other methods on four datasets in the SIR model with γ=0.01. The influential spreaders were set as the initial recovered node.

In practical network applications, such as in malware prevention, the goal is to prevent the spread of malicious information, such as malware, on a large scale in the network, while keeping the defense cost within a limited budget. Therefore, it is necessary to identify the group of nodes with the greatest spreading influence and strengthen their defenses to achieve the smallest final infection scale under limited costs. In this paper’s experiments, we performed an immune operation on the identified influential nodes by setting their initial state as *R*. We then randomly selected a fixed proportion of nodes in the network as infected nodes and observed the final infection scale $P_{r}$. In contrast to the experimental results shown in Figure 3, the smaller the final infection scale $P_{r}$, the better the overall defense effect of selecting this group of nodes. As shown in Figure 5, the SpreadRank method is better than other methods in suppressing the spread of information, which keeps the network at a lower infection level. Moreover, the slope of the curve indicates that the SpreadRank method can effectively suppress the infection scale at a low growth rate, instead of maintaining a high-speed growth rate, when the infection rate is relatively large.

From the above experiments, it can be concluded that the set of influential nodes with the greatest spreading influence obtained by the SpreadRank method proposed in this paper can maximize the infection scale when it is used as an initial infected node. At the same time, when it is used as an immune node, the infection scale can be minimized, thereby effectively preventing the spread of malicious information.

## 4. Conclusions

This paper proposes a novel method, called SpreadRank, for identifying influential spreaders in networks. The method ranks the importance of nodes based on the path reachability between local vital nodes and establishes a dynamic node selection mechanism to obtain a set of nodes with the greatest spread influence. The proposed method is tested on four real networks, and the experimental results demonstrate its effectiveness compared to other existing methods. Additionally, the selected influential node set exhibits superior spread and defense performance when used as an infected or immune node, respectively.

One of the strengths of the SpreadRank method is that it selects local vital nodes as the calculation object, which significantly reduces the computational burden and makes it suitable for large-scale network scenarios. However, the method assumes that there are only a few local vital nodes in scale-free networks, which may not hold for other network topology forms, such as random or hierarchical networks. Future work can address this limitation and explore the effectiveness of SpreadRank in those scenarios.

## Figures and Tables

**Table 1:** Description of indicator parameters.

Factors	Description
Path number (PN)	The number of valid spread paths between two local vital nodes.
Path length (PL)	The average length of the valid spread path between two local vital nodes.
Path diversity (PD)	Diversity of all valid spread paths between two local vital nodes.

**Table 2:** The basic statistics of four real networks. These statistics include the number of nodes (*N*), the number of edges (*E*), the average degree (<*k*>), maximum degree ($k_{max}$), the average clustering coefficient (<*c*>), average distance of the shortest path (<dist>).

Network	*N*	*E*	<*k*>	$k_{\max}$	<*c*>	<dist>
Email	1145	5571	9.04	71	0.2202	3.69
PGP	8846	31,839	7.19	40	0.0072	4.25
Ia-reality	6763	7581	2.37	261	0.0178	4.78
DBLP	12,582	49,630	6.98	710	0.1192	3.81

## Data Availability

All data generated or analyzed during this study are available from the authors on reasonable request.
